# The Possible Precipitating Role of SARS-CoV-2 in a Case of Late-Onset Hemichorea Due to a Hyperosmolar Hyperglycemic State: Case Report and Brief Literature Review

**DOI:** 10.3390/medicina59111949

**Published:** 2023-11-04

**Authors:** Roberto Sperotto, Laura Ceccarelli, Yan Tereshko, Giovanni Merlino, Gian Luigi Gigli, Mariarosaria Valente

**Affiliations:** 1 Clinical Neurology Unit, Udine University Hospital, Piazzale Santa Maria della Misericordia 15, 33100 Udine, Italy; 2 Department of Medicine (DAME), University of Udine, 33100 Udine, Italy

**Keywords:** nonketotic hyperosmolar hyperglycemic state, hyperosmolar hyperglycemic hemichorea, delayed-onset hemichorea, choreic movements, hyperkinetic syndrome, movement disorder, movement disorder and COVID-19

## Abstract

*Case report*: An 83-year-old Italian female developed postural instability and gait disturbance associated with a concomitant hyperosmolar hyperglycemic state. Brain CT and MRI scans detected a lesion in the right putamen due to metabolic derangement. A month later, the patient started suffering from choreic movements along the left side of the body with brachio-crural distribution, approximately three weeks after SARS-CoV-2 infection. She was treated with tetrabenazine with complete resolution of the aberrant movements. Any attempt to reduce tetrabenazine caused a relapse of the symptoms. *Discussion*: In diabetic patients, choreic syndrome should be considered a rare event with a benign prognosis and favorable response to treatment. It is the result of a condition known as “diabetic striatopathy”. The association of new-onset choreic movements, an episode of hyperglycemia, and a basal ganglia lesion is suggestive of this condition. Its pathophysiology remains unclear, and a lot of hypotheses are still debated. SARS-CoV-2 might have played a role in triggering the patient’s motor symptoms. *Conclusions*: Our case report agrees with the general features of those reported in the literature about movement disorders in diabetic patients. The late onset of symptoms and the poor response to treatment seem to be atypical characteristics of the syndrome. Although speculative, we cannot exclude the role of SARS-CoV-2. This case can be added to the literature for further studies and reviews.

## 1. Introduction

Hemichorea is a movement disorder characterized by involuntary and irregular hyperkinetic movements involving one side of the body. This condition is usually associated with contralateral basal ganglia dysfunction, consequent to a lesion within their circuitry. Although uncommon, hyperglycemia is a well-known cause of hemichorea. Its prevalence is higher among elderly female patients of Asian ethnicity, especially with type 2 diabetes and poor control of glycemia [1,2]. This condition is probably underestimated in other populations [3]. Several hypotheses have been proposed so far in terms of pathophysiology, but none explain the process leading to what is called “striatopathy”. The timing of onset, the clinical features, and the response to treatment are not always the same, but, in general terms, the syndrome is characterized by a close correlation with occasional diabetic decompensation, unilateral involvement—but sometimes bilateral—and normalization after glycemic correction and/or with the use of anti-chorea drugs, such as tetrabenazine and antipsychotics [4]. Hereby, we present a case report with some atypical characteristics compared to those most frequently described.

## 2. Case Report (Figure 1)

An 83-year-old Italian woman with no previous personal or familial history of neurologic disorders presented to our outpatient clinic with continuous, involuntary, non-suppressible choreic movements involving the left side of the body. The intrusive movements started in the left upper and lower limbs at the beginning of March 2022 and increased in amplitude and frequency over time. Her past medical history was consistent with dyslipidemia, hypertension, and long-standing type 2 diabetes, complicated by retinopathy, neuropathy, chronic kidney disease, and bilateral carotid stenosis. Importantly, she was not compliant with therapy, particularly with the hypoglycemics prescribed (basal insulin 15 UI + linagliptin 5 mg daily). A few weeks before, on 28 January 2022, she was hospitalized for subacute gait disturbance with postural instability, lateral and retropulsion. At that time, she was diagnosed with a hyperosmolar hyperglycemic state with glycemia around 500 mg/dL and HbA1c of 18%. A head CT scan (Figure 2a) was performed during the hospitalization, and a hyperdense alteration on the right putamen was detected, initially suspected to be neoplastic. A more sophisticated MRI scan was then performed, showing a hyperintense lesion in TSE, TSE-fs, and T1W with negative DW imaging, compatible with the metabolic mismatch.

**Figure 1:**
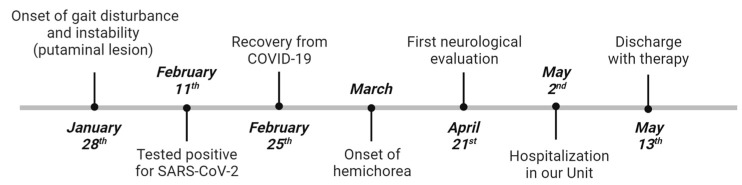
Timeline: summary of the events (created using BioRender).

**Figure 2:**
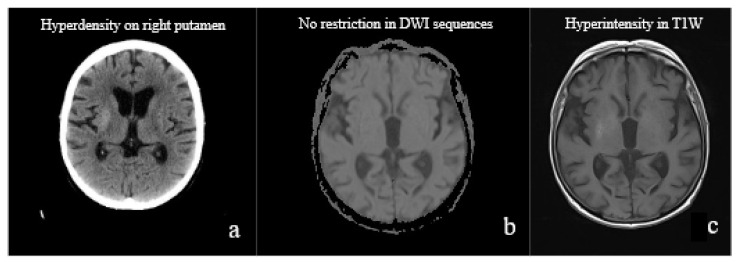
Imaging findings. (**a**) Brain CT (28/01/2022) with evidence of a hyperdense alteration on the right putamen. (**b**,**c**) MRI (10/05/2022) in DWI and T1W sequences with evidence of a persistent hyperintense lesion on the right putamen and no sign of restriction in diffusion sequences.

During the same hospitalization, on 11 February 2022, she contracted SARS-CoV-2 with mild respiratory symptoms and benign evolution. On 25 February, she completely recovered from COVID-19. At the beginning of March, she started suffering from aberrant movements. The first neurological evaluation was performed on 21 April, when hemichorea was diagnosed, and a new short period of hospitalization was suggested. At the time of our evaluation, on 2 May, the neurological examination revealed brachio-crural hemiballismus and hemichorea-like movements. Blood tests were unremarkable. In particular, random glycemia was in range (101 mg/dL), and diabetes was under control throughout the whole hospitalization. An MRI brain scan (Figure 2b,c), in T1W sequences, showed that the hyperintense focal lesion on the right putamen was still detectable, although radiological improvement was pointed out. Cerebrospinal fluid analysis showed only an elevation of pro-inflammatory cytokines and neurofilaments (Table 1), and this was interpreted as the expression of neuronal damage, probably due to the recent metabolic insult and/or to the intercurrent viral infection. Microbiological tests were negative, but eventual positivity for SARS-CoV-2 was not even considered or tested, given the recent recovery from COVID-19. Cerebrospinal fluid and blood onconeural and encephalitis-related antibodies were not detected, and no neoplasm was found. Thus, paraneoplastic syndrome and autoimmune encephalitis were excluded. Neurophysiological tests were also performed. Electroencephalography and polysomnography did not reveal any specific findings, but video recording during night-time showed a complete cessation of movement. Once the investigations were completed, hypoglycemic therapy was not modified and, to better control the motor symptoms, the patient started tetrabenazine 100 mg daily with progressive improvement, up to complete cessation, of the aberrant movements. At the 3-month follow-up, the patient was on tetrabenazine 75 mg daily (reduced for drowsiness) and she was no longer suffering from hemichorea. Still, any attempt at tetrabenazine tapering caused a relapse of symptoms. While preparing this article, we learned that the patient, who was lost to further follow-up, eventually died.

## 3. Brief Review of the Literature

### 3.1. Role of Hyperosmolar Hyperglycemic State

In the literature, several reports of chorea are associated with intercurrent diabetic decompensation. Hyperglycemia may cause choreic symptoms, mainly in elderly female patients of Asian ethnicity [1] with type 2 diabetes, as reported by Bedwell, who first described this syndrome in 1960 [2]. Similar cases are also reported worldwide [3]. The incidence of this condition seems to be very low because it occurs in fewer than 1 in 1,000,000 diabetic patients [5], especially in a setting of poor control of glycemia during—or shortly after—its acute decompensation [6,7,8]. In a broad internal survey of the Mayo Clinic concerning all the cases of chorea and ballismus, hyperglycemia, as the trigger, was discovered in 1% of them [9]. A meta-analysis [5] shows that the mean glycemia at the usual onset of motor symptoms is around 481.5 mg/dL, and HbA1c levels are around 14.4%, proof of miscontrolled diabetes. Some authors describe this syndrome as benign, with favorable evolution in most cases [10]. A cerebral lesion within the circuitry of the basal ganglia may be detected on brain computed tomography (CT) or on an MRI scan. Particularly, on brain CT, the lesion usually appears hyperdense and might be confused with a hemorrhagic phenomenon, whereas on an MRI scan, it appears hyperintense on T1W sequences [11]. The damage to the putamen is more frequent than others described [4] and may persist for a long period of time after onset [4,12,13,14,15]. In most cases, the damage is unilateral and may lead to hemichorea [16]. This condition is known as “diabetic striatopathy”. The pathophysiology of the syndrome remains debated nowadays. There is speculation about changes in cells’ metabolism with a preference for anaerobic energy pathways, impaired neurotransmission, glucotoxicity, ischemia, and microhemorrhages. To date, in patients with dyskinesias, the imaging of SPECT and PET reveals a reduced metabolic rate in the injured basal ganglia. Particularly, spectroscopy demonstrates in-site energy depletion and cellular dysfunction. Diabetic striatopathy might also be linked to precise neuropathological alterations, as well (astrocytosis, gemistocyte formation, inflammatory infiltration, necrosis). In summary, blood hyperviscosity, glucose toxicity, and vasculopathy might lead to neuro-degeneration, thalamic disinhibition and the onset of hyperkinetic disorders, such as chorea [1,12,14,17,18,19,20,21,22]. From a clinical perspective, choreic movements usually involve one side of the body, but more rarely, they can be bilateral. In some cases, the onset of choreic movements occurs in the contralateral side of the body [4]. In some others, they occur ipsilateral to the cerebral lesion [23,24]. The onset of the aberrant movements may be preceded by non-specific symptoms, such as numbness, weakness, gait imbalance, vertigo, and dizziness [4,25]. There is not always a close temporal correlation between diabetic decompensation and motor symptoms, and, to our knowledge, few case reports describe a delayed onset of chorea up to 3 months after the trigger (Table 2), and they seem to be similar to ours [25,26,27,28,29]. In general, these types of movements typically manifest during waking time and cease during sleep, and they tend to normalize after the correction of the glycemia and/or with the use of tetrabenazine and reserpine, which are dopamine-depleting agents; benzodiazepines, enhancing GABA signaling; and/or antipsychotics such as haloperidol (most used), chlorpromazine, sulpiride, tiapride, and pimozide, with variable outcomes [4,5,14,30,31]. Despite the radiological resolution of the cerebral lesion, 20% of cases continue to suffer from recurrent choreic movements and might experience relapse right after stopping anti-chorea drugs in two years after the first episode of movement disorder [4,5]. Symptoms may still resolve spontaneously over a long time, as reported by Salem Amr [32]. Complete resolution might vary from a few days to several months, with a median time of 6 months after the acute event [5].

### 3.2. SARS-CoV-2 and Movement Disorders

SARS-CoV-2 might play a role in the genesis of movement disorders in both hypo- and hyperkinetic phenotypes [33]. As highlighted in the literature, de novo movement disorders have been reported after COVID-19. Mehri Salari et al. stated that the overall frequency of movement disorders in hospitalized COVID-19 patients may be less than 1% [34]. Myoclonus is the most frequently identified, but ataxia, tremor, and choreic movements have also been recently described [35]. Chorea, particularly, may be a rare manifestation of COVID-19, and its pathogenesis is still misunderstood. Hypothetically, there might be multiple routes for SARS-CoV-2 to enter the CNS. Importantly, there is speculation about trans-synaptic pathways, olfactory nerves, hematogenous diffusion, and lymphatic tropism [36]. There is currently no clear evidence regarding the possibility for the virus to infect neuronal cells and, additionally, the description of the isolated virus in CSF is uncommon and controversial [35]. The hypothetical involvement of the nervous system and its functions might be an epiphenomenon of metabolic derangement, with alterations similar to those described during an acute episode of hyperglycemic toxicity, as well as a product of ischemic changes or immunological mechanisms [33,35,37]. However, If the causes involving hyperglycemia are not yet completely understood, those involving SARS-CoV-2 need more study.

## 4. Discussion

Considering the elements provided by the literature and those obtained during hospitalizations, the “metabolic hypothesis” may be considered first. Endocrine disorders, particularly a hyperosmolar hyperglycemic state, have been associated with several neurological symptoms, such as hemichorea, hemiballism, and also epilepsia partialis continua [38]. As reported [4,25], motor disorders may be preceded by disturbances such as gait imbalance, instability, numbness, vertigo, and dizziness, quite non-specific symptoms concerning the damage highlighted on a head CT scan. In our case, too, at first hospitalization, the patient experienced possible prodromic symptoms, which disappeared after the normalization of glycemia. The head CT scan performed in the Emergency Department showed the presence of a hyperdense lesion within the right basal ganglia, which, in further MRI scan investigations, was linked to prior metabolic derangement. The core features of this kind of lesion are superimposable to those described in [11]; particularly, they are frequently hyperintense in T1W sequences and DWI-negative. New-onset motor disorders were not reported from the available medical charts related to the first hospitalization. Effectively, the late onset of a hyperkinetic disturbance seems to be unusual; however, reports exist describing cases that are similar to ours in which the hyperkinetic syndrome appears in the context of glycemic control, after the acute event. Moreover, as described in Table 2, the motor symptomatology might develop up to several months after the trigger. From an epidemiological point of view, the illustrated syndrome affects the type of patient described in the most important reports and reviews. These are elderly female patients with poor control of type 2 diabetes [1,2]. Moreover, the fact that the symptoms were lateralized agrees with the lesion and its site, for the aberrant movements appeared contralaterally. Thus, it is plausible to suppose that they depend on the lesion itself, rather than on a new kind of damage, possibly caused by COVID-19. As can be seen, multiple elements support the first hypothesis of a metabolic origin. However, as underlined in the description of the events, the “particularity” of our case might concern the intercurrent SARS-CoV-2 infection, which, in our opinion, could have somehow contributed to the onset or activation of motor symptoms with pathomechanisms not yet fully clarified, given the close temporal correlation between events (infection and appearance of hemichorea) and the possibility, described in the literature, of developing movement disorders. In this case, what made us suspect a possible intervention of SARS-CoV-2 in the development of motor symptoms was certainly the reports available in the literature, but also the peculiar cytokine panel, which showed an active inflammatory state associated with non-specific neuronal degeneration (represented by an increase in neurofilaments), despite a radiological improvementwas pointed out at 3 months from the hyperglycemic insult. We cannot confirm or exclude that this cytokine panel may or may not be linked to previous metabolic damage, as we have no available comparisons. Moreover, we cannot know whether SARS-CoV-2 could also have contributed to the cytokine elevation observed during the second hospitalization. What we can empirically suspect is that the presence of any brain insult (metabolic or infectious) leads to an increase in neurofilaments and in inflammatory cytokines, with a shift towards a pro-inflammatory setting, which can be prolonged in time, or not, depending on the system’s capacity to clear the inflammation. To date, our opinion remains a hypothesis that might not be confirmed, except by other speculation in the literature, that requires further methodological investigations. The most probable hypothesis remains the metabolic one, as it is better supported by evidence from the literature in cases that most closely resemble ours. However, we do not wish to exclude a possible pathogenetic role of SARS-CoV-2 infection, which could have acted on an already weakened cerebral site, triggering motor symptoms later in time.

## 5. Conclusions

In diabetic patients, hemichorea should be considered a rare event with a general benign prognosis and favorable response to treatment. It is the result of a condition known as “diabetic striatopathy”, which is characterized by the association of new-onset choreic movements, hyperglycemia, and a basal ganglia lesion. The late onset of symptoms and the poor response to treatment seem to be atypical or uncommon characteristics of an already rare syndrome. The relapse of symptoms during tetrabenazine tapering, although potentially suggestive of a prolonged “metabolic” syndrome, cannot be seen as proof of permanent hemichorea, since a better outcome over time cannot be ruled out, given the limited follow-up period. Moreover, in consideration of the current state of knowledge, it cannot be completely ruled out that the superimposition of the cerebral inflammatory state, documented by cytokine elevation in the cerebrospinal fluid analysis, potentially related to the intercurrent COVID-19, might have contributed to triggering extrapyramidal movements several weeks after the radiological evidence of “diabetic striatopathy”.

## Figures and Tables

**Table 1:** Analysis of some detectable cytokines in cerebrospinal fluid with evidence of active inflammation 3 months after the onset of the lesion on brain CT.

Cytokine Type	Detection	Normal Range
IL-15	9.3 pg/mL	1.9–2.6 pg/mL
IL-6	3.3 pg/mL	1–3.1 pg/mL
IL-8	94.2 pg/mL	15.2–38.4 pg/mL
TNFα	1.5 pg/mL	<0.5 pg/mL
CXCL10/IP10	633 pg/mL	6–132 pg/mL
IL-2 receptor α	148 pg/mL	5.7–19.4 pg/mL
Neurofilaments	2570 pg/mL	62–514 pg/mL

**Table 2:** To our knowledge, these are the reported cases of late-onset hemichorea after glycemic decompensation in patients affected by diabetes mellitus (DM).

Name of the Study	Gender, Age	Risk Factors	Glycemia at First Evaluation	Symptoms at First Evaluation	Late-Onset Hemichorea/Hemiballism	Treatment	Outcome
**Abe et al. [25]**	M, 73 yrs	DM (not specified), hypertension	151 mg/dL HbA1c 17.2%	Numbness in right upper and lower limbs	2 weeks later	Haloperidol	Improvement
**Abe et al. [25]**	M, 16 yrs	DM 1	312 mg/dL HbA1c 17.9%	Numbness in left upper and lower limbs	A month later	Haloperidol	Improvement
**Abe at al. [25]**	W, 84 yrs	DM (not specified), hypertension	107 mg/dL HbA1c 7.4% (previous 12.3%)	Transient weakness of right hand	10 days later	Haloperidol	Improvement
**Lin, Huang [26]**	M, 73 yrs	DM (not specified)	815 mg/dL HbA1c 17.3%	Episode of non-ketotic hyperglycemia (symptoms not specified)	A month later	Haloperidol, clonazepam	Progressive resolution
**Bizet et al. [27]**	W, 66 yrs	DM 2, hypertension, dyslipidemia	984 mg/dL	Episode of non-ketotic hyperglycemia (symptoms not specified)	3 months later	Risperidone	Resolution
**Aguiar et al. [28]**	W, 69 yrs	DM (not specified)	Not available	Episode of non-ketotic hyperglycemia (symptoms not specified)	2 weeks later	Not available	Not available
**Lim et al. [29]**	W, 70 yrs	DM 2	46 mmol/L HbA1c 16.3%	Lethargy and confusion	2 weeks later	Tetrabenazine	Improvement

## Data Availability

Not applicable.

## References

[1] Hsu J.L., Wang H.C., Hsu W.C. (2004). Hyperglycemia-induced unilateral basal ganglion lesions with and without hemichorea A PET study. J. Neurol..

[2] Bedwell S.F. (1960). Some observations on hemiballismus. Neurology.

[3] Cosentino C., Torres L., Nuñez Y., Suarez R., Velez M., Flores M. (2016). Hemichorea/Hemiballism Associated with Hyperglycemia: Report of 20 Cases. Tremor Other Hyperkinetic Mov..

[4] Chua C.B., Sun C.K., Hsu C.W., Tai Y.C., Liang C.Y., Tsai I.T. (2020). “Diabetic striatopathy”: Clinical presentations, controversy, pathogenesis, treatments, and outcomes. Sci. Rep..

[5] Oh S.H., Lee K.Y., Im J.H., Lee M.S. (2002). Chorea associated with non-ketotic hyperglycemia and hyperintensity basal ganglia lesion on T1-weighted brain MRI study: A meta-analysis of 53 cases including four present cases. J. Neurol. Sci..

[6] Lin J.J., Chang M.K. (1994). Hemiballism-hemichorea and non-ketotic hyperglycaemia. J. Neurol. Neurosurg. Psychiatry.

[7] Rector W.G., Herlong H.F., Moses H. (1982). Nonketotic hyperglycemia appearing as choreoathetosis or ballism. Arch. Intern. Med..

[8] Kataja Knight A., Magnusson P., Sjöholm Å. (2021). Hemiballismus in hyperglycemia. Clin. Case Rep..

[9] Ifergane G., Masalha R., Herishanu Y.O. (2001). Transient hemichorea/hemiballismus associated with new onset hyperglycemia. Can. J. Neurol. Sci..

[10] Lee B.C., Hwang S.H., Chang G.Y. (1999). Hemiballismus-hemichorea in older diabetic women: A clinical syndrome with MRI correlation. Neurology.

[11] Hegde A.N., Mohan S., Lath N., Lim C.C. (2011). Differential diagnosis for bilateral abnormalities of the basal ganglia and thalamus. Radiographics.

[12] Chu K., Kang D.W., Kim D.E., Park S.H., Roh J.K. (2002). Diffusion-weighted and gradient echo magnetic resonance findings of hemichorea-hemiballismus associated with diabetic hyperglycemia: A hyperviscosity syndrome?. Arch. Neurol..

[13] Iwata A., Koike F., Arasaki K., Tamaki M. (1999). Blood brain barrier destruction in hyperglycemic chorea in a patient with poorly controlled diabetes. J. Neurol. Sci..

[14] Shan D.E., Ho D.M., Chang C., Pan H.C., Teng M.M. (1998). Hemichorea-hemiballism: An explanation for MR signal changes. AJNR Am. J. Neuroradiol..

[15] Massaro F., Palumbo P., Falcini M., Zanfranceschi G., Pratesi A. (2012). Generalized chorea-ballism in acute non ketotic hyperglycemia: Findings from diffusion-weighted magnetic resonance imaging. Parkinsonism Relat. Disord..

[16] Battisti C., Forte F., Rubenni E., Dotti M.T., Bartali A., Gennari P., Federico A., Cerase A. (2009). Two cases of hemichorea-hemiballism with nonketotic hyperglycemia: A new point of view. Neurol. Sci..

[17] Postuma R.B., Lang A.E. (2003). Hemiballism: Revisiting a classic disorder. Lancet Neurol..

[18] Hashimoto K., Ito Y., Tanahashi H., Hayashi M., Yamakita N., Yasuda K. (2012). Hyperglycemic chorea-ballism or acute exacerbation of Huntington’s chorea? Huntington’s disease unmasked by diabetic ketoacidosis in type 1 diabetes mellitus. J. Clin. Endocrinol. Metab..

[19] Duckrow R.B., Beard D.C., Brennan R.W. (1985). Regional cerebral blood flow decreases during hyperglycemia. Ann. Neurol..

[20] Lee E.J., Choi J.Y., Lee S.H., Song S.Y., Lee Y.S. (2002). Hemichorea-hemiballism in primary diabetic patients: MR correlation. J. Comput. Assist. Tomogr..

[21] Kim J.S., Lee K.S., Lee K.H., Kim Y.I., Kim B.S., Chung Y.A., Chung S.K. (2002). Evidence of thalamic disinhibition in patients with hemichorea: Semiquantitative analysis using SPECT. J. Neurol. Neurosurg. Psychiatry.

[22] Lin J.J., Lin G.Y., Shih C., Shen W.C. (2001). Presentation of striatal hyperintensity on T1-weighted MRI in patients with hemiballism-hemichorea caused by non-ketotic hyperglycemia: Report of seven new cases and a review of literature. J. Neurol..

[23] Lin J.J. (2001). Ipsilateral putamen hyperintensity on T1-weighted MRI in non-ketotic hyperglycemia with hemiballism-hemichorea: A case report. Parkinsonism Relat. Disord..

[24] Fong S.L., Tan A.H., Lau K.F., Ramli N., Lim S.Y. (2019). Hyperglycemia-Associated Hemichorea-Hemiballismus with Predominant Ipsilateral Putaminal Abnormality on Neuroimaging. J. Mov. Disord..

[25] Abe Y., Yamamoto T., Soeda T., Kumagai T., Tanno Y., Kubo J., Ishihara T., Katayama S. (2009). Diabetic striatal disease: Clinical presentation, neuroimaging, and pathology. Intern. Med..

[26] Lin C.J., Huang P. (2017). Delayed onset diabetic striatopathy: Hemichorea-hemiballism one month after a hyperglycemic episode. Am. J. Emerg. Med..

[27] Bizet J., Cooper C.J., Quansah R., Rodriguez E., Teleb M., Hernandez G.T. (2014). Chorea, Hyperglycemia, Basal Ganglia Syndrome (C-H-BG) in an uncontrolled diabetic patient with normal glucose levels on presentation. Am. J. Case Rep..

[28] Aguiar T., Nogueira R., Vidon R., Silva M.M., Maranhão-Filho P. (2013). Delayed hemichorea syndrome associated with nonketotic hyperglycemia. Arq. Neuropsiquiatr..

[29] Lim K.X., Khaing Zin T., Yu Z., Peh W.M. (2022). Delayed Presentation of Hemichorea in Diabetic Striatopathy. Cureus.

[30] Ondo W.G. (2011). Hyperglycemic nonketotic states and other metabolic imbalances. Handb. Clin. Neurol..

[31] Ahlskog J.E., Nishino H., Evidente V.G., Tulloch J.W., Forbes G.S., Caviness J.N., Gwinn-Hardy K.A. (2001). Persistent chorea triggered by hyperglycemic crisis in diabetics. Mov. Disord..

[32] Salem A., Lahmar A. (2021). Hemichorea-Hemiballismus Syndrome in Acute Non-ketotic Hyperglycemia. Cureus.

[33] Schneider S.A., Hennig A., Martino D. (2022). Relationship between COVID-19 and movement disorders: A narrative review. Eur. J. Neurol..

[34] Salari M., Zaker Harofteh B., Etemadifar M., Sedaghat N., Nouri H. (2021). Movement Disorders Associated with COVID-19. Parkinsons Dis..

[35] Brandão P.R.P., Grippe T.C., Pereira D.A., Munhoz R.P., Cardoso F. (2021). New-Onset Movement Disorders Associated with COVID-19. Tremor Other Hyperkinet Mov..

[36] Sepehrinezhad A., Shahbazi A., Negah S.S. (2020). COVID-19 virus may have neuroinvasive potential and cause neurological complications: A perspective review. J. Neurovirol..

[37] Crunfli F., Carregari V.C., Veras F.P., Vendramini P.H., Valença A.G.F., Antunes A.S.L.M., Brandão-Teles C., da Silva Zuccoli G., Reis-de-Oliveira G., Silva-Costa L.C. (2020). SARS-CoV-2 infects brain astrocytes of COVID-19 patients and impairs neuronal viability. medRxiv.

[38] Cruz-Flores S. (2021). Neurological complications of endocrine emergencies. Curr. Neurol. Neurosci. Rep..

